# Pulmonary Thrombosis Promotes Tumorigenesis via Myeloid Hypoxia-Inducible Factors

**DOI:** 10.3390/biom12101354

**Published:** 2022-09-23

**Authors:** Xiao Lu, Alice Prodger, Jingwei Sim, Colin E. Evans

**Affiliations:** 1 Department of Physiology, Development and Neuroscience, University of Cambridge, Cambridge CB2 3EG, UK; 2 British Heart Foundation Centre of Research Excellence, University of Cambridge, Cambridge CB2 3EG, UK

**Keywords:** HIFs, hypoxia, microvascular, thrombosis, tumorigenesis

## Abstract

Cancer patients have a greater risk of thrombosis than individuals without cancer. Conversely, thrombosis is a diagnostic predictor of cancer, but the mechanisms by which thrombosis promotes tumor propagation are incompletely understood. Our previous studies showed that hypoxia-inducible factors (HIF) 1α and HIF2α are stabilized in myeloid cells of murine thrombi. We also previously showed that pulmonary thrombosis increases the levels of HIF1α and HIF2α in murine lungs, enhances the levels of tumorigenic factors in the circulation, and promotes pulmonary tumorigenesis. In this study, we aimed to investigate the regulation of thrombosis-induced tumorigenesis by myeloid cell-specific HIFs (i.e., HIF1 and HIF2 in neutrophils and macrophages). Our in vitro studies showed that multiple tumorigenic factors are upregulated in the secretome of hypoxic versus normoxic neutrophils and macrophages, which promotes lung cancer cell proliferation and migration in a myeloid-HIF-dependent manner. Next, we used a mouse model of pulmonary microvascular occlusion to study the impact of pulmonary thrombosis and myeloid HIFs on lung tumorigenesis. Experiments on mice lacking either HIF1α or HIF2α in myeloid cells demonstrated that loss of either factor eliminates the advantage given to pulmonary tumor formation by thrombotic insult. The myeloid HIF-dependent and tumorigenic impact of pulmonary thrombosis on tumor burden may be partly driven by paracrine thymidine phosphorylase (TP), given that TP levels were increased by hypoxia in neutrophil and macrophage supernates, and that plasma TP levels were positively correlated with multiple measures of tumor progression in wild type mice but not myeloid cell-specific HIF1α or HIF2α knockout mice. These data together demonstrate the importance of thrombotic insult in a model of pulmonary tumorigenesis and the essential role of myeloid HIFs in mediating tumorigenic success.

## 1. Introduction

Thrombosis and thromboembolism are independent predictors of cancer [1,2]. Conversely, the incidence of thrombosis is increased in a variety of cancer types (including lung cancer) compared with healthy individuals, and in metastatic compared with non-metastatic cancer patients [3]. Anti-coagulants protect against pulmonary metastasis, while thrombotic agents have the opposite effect [4,5,6,7], but the mechanisms by which thrombosis promotes tumor propagation are incompletely understood. A better understanding of the mechanisms that control thrombosis-associated cancer progression could impact upon treatment strategies for cancer patients, particularly in those with concurrent thromboembolic disease.

Hypoxia is a major characteristic of many solid tumor types [8], and murine thrombi are hypoxic compared with venous blood [9]. The vascular remodelling response to hypoxia is controlled by the hypoxia-inducible factors (HIFs) 1 and 2 [10]. These transcription factors are composed of a constitutively-expressed β subunit and a hypoxia-stabilized α subunit [10]. Downstream target genes of HIF1 and HIF2 are distinct but overlapping and include many pro-angiogenic and pro-tumorigenic cytokines and growth factors [11,12]. The expression of HIF1α and HIF2α is stabilised in myeloid cells (i.e., neutrophils and macrophages) contained within murine thrombi [9,13,14]. Notably, the remodelling response that follows occlusive thrombus formation is pro-angiogenic, and is preceded by increases in neutrophil HIF1α and macrophage HIF2α [9,13,15]. We previously used a model of pulmonary microvascular occlusion to show that the induction of pulmonary microthrombosis enhances pulmonary expression of HIF1α and HIF2α, and promotes pulmonary tumorigenesis in wild type mice [14]. However, the role of myeloid cell-specific HIFs in thrombosis-induced tumor propagation is unknown. Given that myeloid cell hypoxia and HIF signaling is integral to the processes of thrombus formation [16,17], thrombus resolution [9,18], and tumorigenesis [8,11,19], we set out to assess the contribution of myeloid cell HIFs to thrombosis-associated tumorignesis in murine lungs.

The primary aim of this study was to determine whether thrombus formation in the pulmonary microvasculature affects pulmonary tumor progression via myeloid cell-specific HIFs. We show that multiple tumorigenic factors are released by hypoxic myeloid cells in a HIF-dependent manner. We also show here that pulmonary thrombosis enhances tumorigenesis in a manner that is dependent upon myeloid cell HIFs 1 and 2 and that thrombosis-induced tumorigenesis seems to be regulated by paracrine factors such as thymidine phosphorylase (TP).

## 2. Materials and Methods

### 2.1. Mice

Myeloid cell-specific *Hif1α* or *Hif2α* knockout (HIF1α or HIF2α KO) mice and wildtype (WT) littermates (8–10 week-old males) were generated on a C57BL/6J background as described [20,21,22]. Animal studies were performed under the Animals (Scientific Procedures) Act, 1986.

### 2.2. Cell Culture

Lewis lung cancer cells (LLCs) were cultured in Dulbecco’s modified eagle medium with 10% fetal bovine serum and 1% penicillin/streptavidin (Life Technologies, Cramlington, UK) at 21% oxygen and 37 °C. WT and *Hifα* KO (HIF1α or HIF2α KO) macrophages were isolated from mouse bone marrow as described [21] and cultured in Dulbecco’s modified eagle medium (Life Technologies, UK) for 24 h at 21% or 1% oxygen and 37 °C. WT and *Hifα* KO (HIF1α or HIF2α KO) neutrophils were isolated from mouse blood as described [22] and cultured in Roswell Park Memorial Institute 1640 medium (Life Technologies, Cramlington, UK) for 14 h at 21% or 1% oxygen and 37 °C. Cell supernates were collected and immediately frozen at −80 °C until use.

### 2.3. Mouse Model of Thrombosis-Associated Tumorigenesis

For the mouse studies (*n* = 10/group), pulmonary thrombosis was induced by intra-venous injection of polystyrene microbeads (15 μm diameter, 1000 beads/mouse in 100 μL saline, Life Technologies, Cramlington, UK) at 1 day after administration of LLCs (1 million/mouse) as described [14]. Mice were culled at day 14 post-LLC injection as described [23].

### 2.4. Histology and Immunostaining

Lungs were fixed in 4% paraformaldehyde and processed then embedded in paraffin using an automated tissue processor (Leica, London, UK). Cross-sections (7 μm) were cut at 200 μm intervals throughout the lung using a manual microtome (Leica, London, UK). Contiguous sections were stained with haematoxylin and eosin (H&E), and for HIF1α, HIF2α, NIMP R14, Mac2, Annexin V, CD34, and proliferating cell nuclear antigen (PCNA) as described [9,24,25,26].

### 2.5. Image Capture and Analysis

Images were captured in a blinded fashion using a brightfield light microscope and mounted camera (Leica, London, UK). Primary antibody binding was assessed by quantification of positive staining using Image J (NIH, Bethesda, MD, USA) as described [9,24,25,26]. Pulmonary tumor burden was assessed as described [23]. Tumor burden values are mean number of foci per lung cross section and mean % of lung cross sectional area containing foci. Immunostaining values are mean % of tumor area covered by positive staining. Pulmonary tumor vascularisation was assessed using a Chalkley reticule as described [27]. Values are mean number of tumor vessels.

### 2.6. Protein Arrays

A panel of 53 tumorigenic growth factors and cytokines were measured in normoxic and hypoxic myeloid cell supernates (*n* = 4/group) by protein array according to manufacturers’ instructions (R&D Systems, London, UK). Values are mean pixel density expressed as % positive control.

### 2.7. Migration and Proliferation Assays

Migration of GFP-labelled LLCs exposed to myeloid cell (neutrophil or macrophage) supernates was assessed by Oris™ Cell Migration Assays according to manufacturers’ instructions (Platypus Technologies, Hove, UK) (*n* = 4/group). The assays were performed at 21% oxygen and 37 °C. Neutrophil supernates were taken from 80% confluent neutrophils that had been exposed to 21% (normoxia, Nx) or 1% (hypoxia, Hx) oxygen for 14 h. Macrophage supernates were taken from 80% confluent macrophages that had been exposed to 21% (normoxia, Nx) or 1% (hypoxia, Hx) oxygen for 24 h. In brief, Oris™ Cell Seeding Stoppers in a 96-well plate restrict cell seeding to the outer annular regions of the wells. When the stoppers are removed, a 2 mm diameter unseeded circular region is revealed in the centre of each well; this is the detection zone into which the seeded GFP-labelled cells can migrate. Subsequently, an Oris™ Detection Mask is applied to the areas of the wells that are outside of the detection zones, which restricts the fluorescence detection to only the cells that have migrated to the detection zone. Data are expressed as area under curve of fluorescence detection, indicative of the number of cells that migrated into the detection zone.

To assess proliferation, equal numbers of LLCs were seeded in 48-well plates and exposed to myeloid cell (neutrophil or macrophage) supernates for 7 days at 21% oxygen and 37 °C (*n* = 4/group). After 7 days incubation, LLCs were washed 3 times with PBS, trypsinized, and counted using an automated cell counter. Cell numbers were confirmed manually with a hemocytometer. To generate myeloid cell (neutrophil or macrophage) supernates, 80% confluent neutrophils were exposed to 21% (normoxia, Nx) or 1% (hypoxia, Hx) oxygen for 14 h, while 80% confluent macrophages were exposed to 21% (normoxia, Nx) or 1% (hypoxia, Hx) oxygen for 24 h. Data are expressed as fold change of the number of seeded LLCs.

### 2.8. Statistical Analysis

The interaction between genotype and treatment was assessed using 2-way analysis of variance (ANOVA) and differences between groups were assessed with Bonferroni post-tests. Correlations were assessed using Pearson’s correlation. Significance was assumed at *p* < 0.05 in all analyses (Prism 5, Graphpad, San Diego, CA, USA). Data are expressed as means +/− standard error.

## 3. Results

### 3.1. Hypoxia Increases the Release of Tumorigenic Factors from Myeloid Cells in a HIF-Dependent Manner

**Figure 1:**
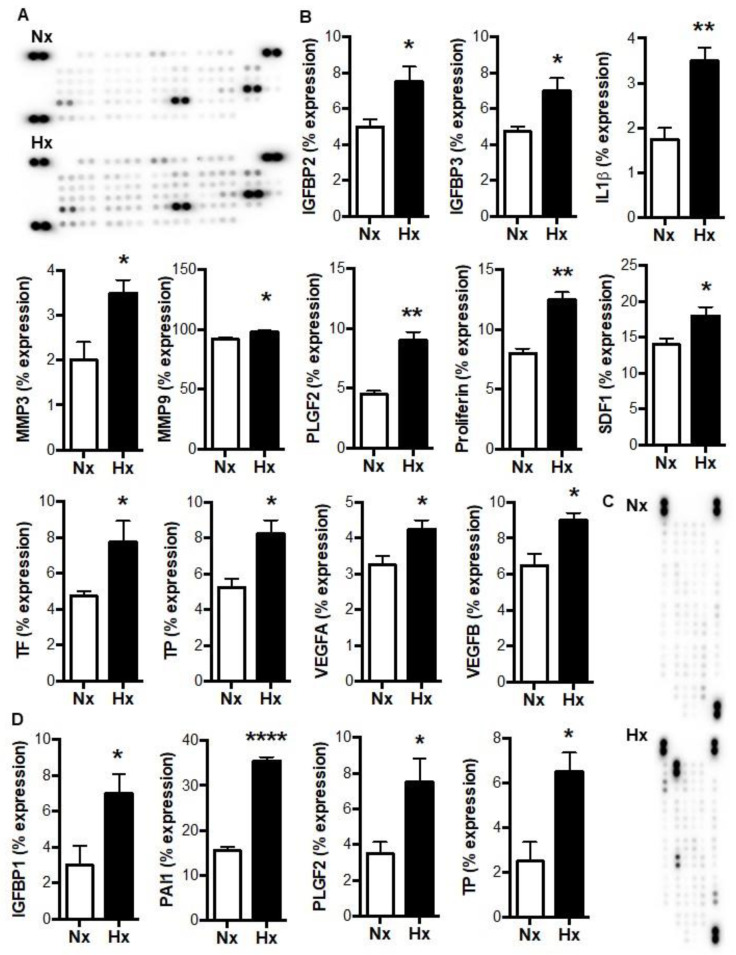
Tumorigenic factors in myeloid cell supernates are increased by hypoxia. (**A**) Representative images and (**B**) quantifications of angiogenic protein array expression performed on neutrophil supernates following 14 h incubation at 21% (normoxia, Nx) or 1% (hypoxia, Hx) oxygen. (**C**) Representative images and (**D**) quantifications of angiogenic protein array expression performed on macrophage supernates following 14 h incubation at 21% (normoxia, Nx) or 1% (hypoxia, Hx) oxygen. *N* = 4–5/group. * *p* < 0.05, ** *p* < 0.01, and **** *p* < 0.0001.

In our previous study of tumor-free mice, pulmonary thrombosis gave rise to increases in lung tumorigenesis, along with increased pulmonary levels of HIF1α and HIF2α, and increases in the circulating levels of multiple tumorigenic factors, including insulin-like growth factor binding protein (IGFBP) 1, interleukin (IL) 1α, IL1β, and TP (formerly known as platelet-derived endothelial cell growth factor, PD-ECGF) [14]. Notably, these 4 factors are all hypoxia-responsive and expressed by myeloid cells [28,29,30,31]. We therefore wished to determine whether increases in the release of tumorigenic factors could be stimulated by the exposure of by myeloid cells to hypoxia, which occurs within murine thrombi [9,14]. As shown in Figure 1A,B, hypoxia increased the levels of 12 different angiogenic/tumorigenic factors in neutrophil supernates. These hypoxia-induced factors included IL1β and TP, as well as known HIF targets such as stromal cell-derived factor 1 and vascular endothelial growth factor (VEGF) A. In macrophage supernates, hypoxia resulted in increased levels of IGFBP1 and TP, along with increased levels of the HIF targets, plasminogen activator inhibitor 1 and placental growth factor 2 (Figure 1C,D). These data suggest that the release of tumorigenic factors by myeloid cells is increased by a level of hypoxia that is comparable to that found in experimental venous thrombi [9].

### 3.2. Paracrine Regulation of Lung Cancer Cell Migration and Proliferation by Myeloid HIFs

**Figure 2:**
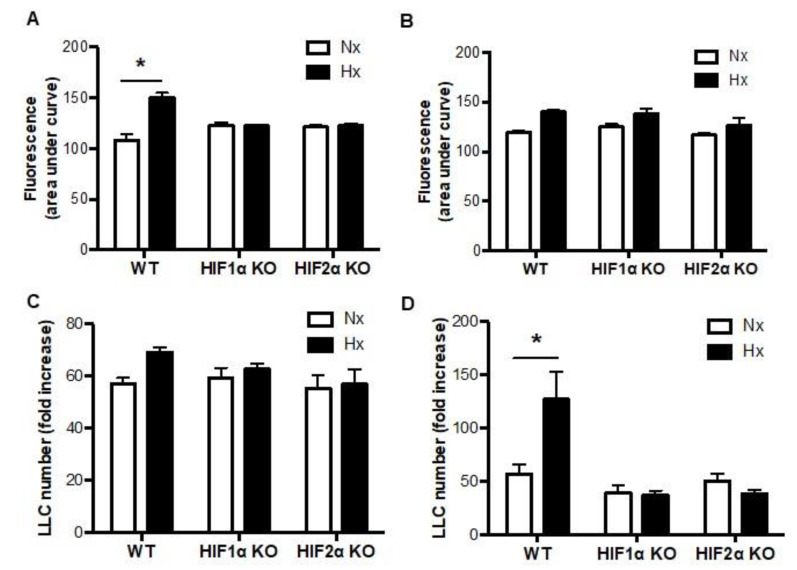
Paracrine regulation of lung cancer cell migration and proliferation by myeloid HIF1α and myeloid HIF2α. (**A**) Migration of LLCs was assessed when exposed to the supernate of WT, HIF1α null, or HIF2α null neutrophils or (**B**) macrophages that had been incubated at 21% (normoxia, Nx) or 1% (hypoxia, Hx) oxygen for 14 or 24 h respectively. The assays were performed under normoxia. (**C**) LLC proliferation was quantified when exposed to the supernate of WT, HIF1α null, or HIF2α null neutrophils or (**D**) macrophages that had been incubated at 21% (normoxia, Nx) or 1% (hypoxia, Hx) oxygen for 14 or 24 h respectively. The assays were performed under normoxia. *N* = 4/group. * *p* < 0.05.

To determine whether myeloid cell HIFs could regulate lung cancer cell tumorigenicity in a paracrine manner, we next exposed LLCs to normoxic and hypoxic myeloid cell supernates, and assessed LLC migration and proliferation. LLC migration was increased when LLCs were exposed to the supernate of hypoxic versus normoxic WT neutrophils (Figure 2A). Hypoxia-induced increases in LLC migration were completely absent, however, when LLCs were incubated in supernate from HIF1α null or HIF2α null neutrophils (Figure 2A). Conversely, there were no hypoxia-induced increases in LLC migration when macrophage supernates were exposed to the LLCs, regardless of the presence or absence of HIF1α or HIF2α in the macrophages (Figure 2B). Next, we showed that there were no hypoxia-induced increases in LLC proliferation when neutrophil supernates were exposed to the LLCs, regardless of the presence or absence of HIF1α or HIF2α in the neutrophils (Figure 2C). LLC proliferation was increased, however, when these cells were cultured in supernate from WT macrophages that had been exposed to hypoxia compared with normoxia, and these hypoxia-induced increases were absent when the LLCs were exposed to supernate from HIF1α or HIF2α null macrophages (Figure 2D). These data suggest that LLC migration is enhanced in a paracrine manner by HIFs 1 and 2 in neutrophils, while LLC proliferation is promoted in a paracrine manner by HIFs 1 and 2 in macrophages.

### 3.3. Pulmonary Microvascular Occlusion Increases Pulmonary Tumorigenesis via Myeloid HIFs

**Figure 3:**
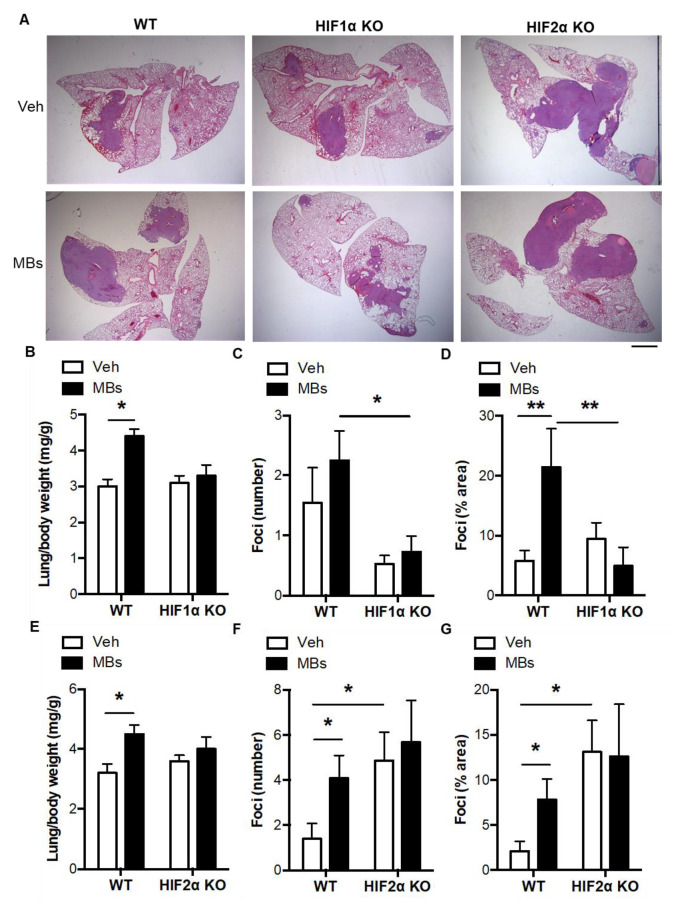
Thrombosis-induced increases in pulmonary tumorigenesis are absent in myeloid HIF1α KO or myeloid HIF2α KO mice. (**A**) Representative images of H&E-stained lung cross sections following intravenous microbead administration in WT, myeloid HIF1α KO mice, and myeloid HIF2α KO mice. Nucleated cell-dense tumor foci are colored purple. Scale bar = 1 mm. (**B**) Lung to body weight ratio, (**C**) pulmonary foci number, and (**D**) pulmonary foci area following intravenous microbead administration in WT and myeloid HIF1α KO mice. (**E**) Lung to body weight ratio, (**F**) pulmonary foci number, and (**G**) pulmonary foci area following intravenous microbead administration in WT and myeloid HIF2α KO mice. Vehicle (Veh) or microbeads (MBs) were administered at 1 day after LLCs. Mice were culled at day 14 post-LLCs. *N* = 10/group. * *p* < 0.05 and ** *p* < 0.01.

Since pulmonary microvascular occlusion would be expected to involve both myeloid cell and HIF response, and given that occlusive venous thrombosis leads to the upregulation of HIF1α and HIF2α in myeloid cells [9,13], we next examined the impact of both myeloid HIF1α and myeloid HIF2α in a mouse model of thrombosis-associated pulmonary tumorigenesis [14]. In wildtype mice, the induction of pulmonary thrombosis by intravenous polystyrene microbeads gave rise to increases in lung to body weight ratio, pulmonary tumor number, and tumor area (Figure 3A–G), consistent with our previous study [14]. Microbead-induced increases in tumor burden were absent, however, in both myeloid HIF1α KO mice and myeloid HIF2α KO mice (Figure 3A–G). Although the microbead-induced pro-tumorigenic effects were eliminated by conditional deletion of either HIFα isoform, there appeared to be an overall difference in the effect of the loss of the 2 HIFα isoforms per se on tumor number and coverage, with a greater tumor burden seen in HIF2α KO mice versus WT and possibly in WT versus HIF1α KO mice (Figure 3C,F,G), consistent with previous studies of lung [23] and intestinal tumorigenesis [19].

**Figure 4:**
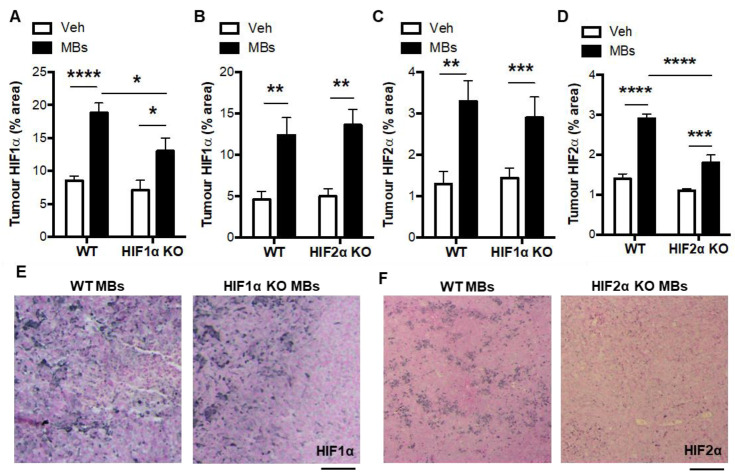
HIFα expression in pulmonary tumors of vehicle- and microbead-treated WT and myeloid HIFα KO mice. (**A**) Pulmonary tumor HIF1α levels following administration of intravenous vehicle (Veh) or microbeads (MBs) at 1 day post-LLCs in WT mice and myeloid HIF1α KO or (**B**) HIF2α KO littermates. (**C**) Pulmonary tumor HIF2α levels following administration of intravenous vehicle (Veh) or microbeads (MBs) at 1 day post-LLCs in WT mice and myeloid HIF1α KO or (**D**) HIF2α KO littermates. (**E**) Representative images of pulmonary tumor sections with immunostaining (black) for HIF1α and (**F**) HIF2α. Scale bar = 50 μm. Mice were culled and lung tissue excised for analysis at day 14 post-LLCs. *N* = 10/group. * *p* < 0.05, ** *p* < 0.01, *** *p* < 0.001, and **** *p* < 0.0001.

**Figure 5:**
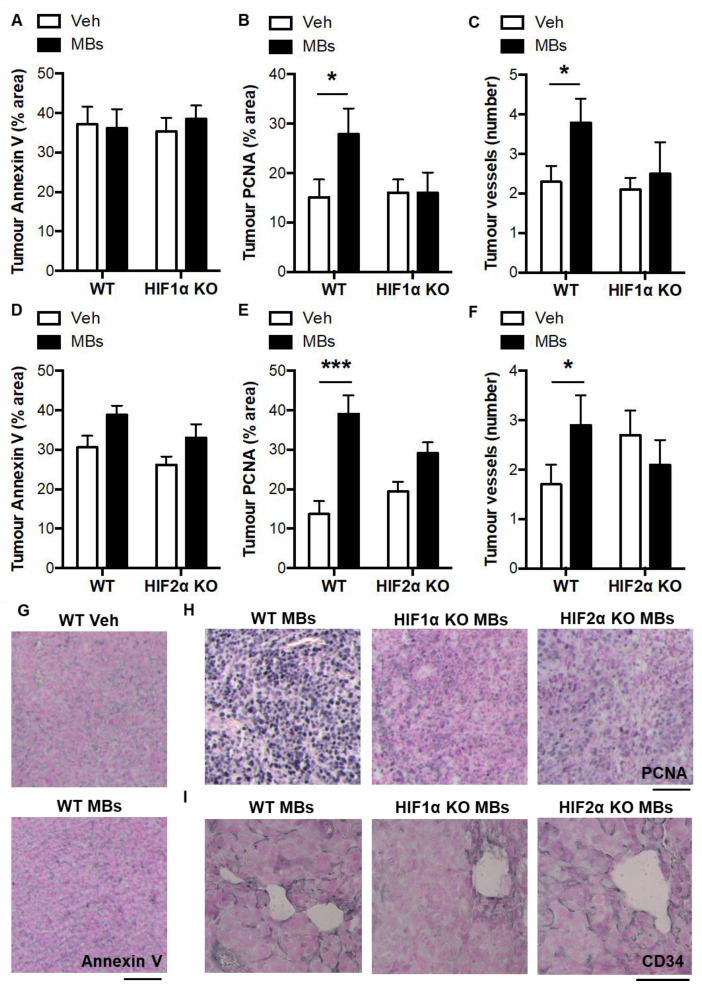
Characterization of pulmonary tumors from vehicle- and microbead-treated WT and myeloid HIFα KO mice. (**A**) Pulmonary tumor apoptosis, (**B**) proliferation, and (**C**) vascularisation in pulmonary tumors following administration of intravenous vehicle (Veh) or microbeads (MBs) at 1 day post-LLCs in WT mice and myeloid HIF1α KO littermates. (**D**) Pulmonary tumor apoptosis, (**E**) proliferation, and (**F**) vascularisation in pulmonary tumors following administration of intravenous vehicle (Veh) or microbeads (MBs) at 1 day post-LLCs in WT mice and myeloid HIF1α KO littermates. Panels (**A**,**B**,**D**,**E**) are quantifications of the percentage area of the pulmonary tumor that was occupied by positive staining for annexin V (apoptosis) or PCNA (proliferation). Quantifications were performed by image analysis. In panels C and F, the number of tumor vessels was counter using a Chalkley reticule and normalized to tumor surface area. (**G**) Representative images of pulmonary tumor sections with immunostaining (black) for Annexin V, (**H**) PCNA, and (**I**) CD34. Scale bar = 50 μm. Mice were culled and lung tissue excised for analysis at day 14 post-LLCs. *N* = 10/group. * *p* < 0.05 and *** *p* < 0.001.

In the lung tumors, HIF1α and HIF2α levels were increased by microbead treatment in WT mice, HIF1α KO mice, and HIF2α KO mice (Figure 4A–D). There were reductions in the tumor HIF1α levels in the microbead-treated HIF1α KO mice compared with the microbead-treated WT mice (Figure 4A,E). Similarly, there were reductions in the tumor HIF2α levels in the microbead-treated HIF2α KO mice compared with the microbead-treated WT mice (Figure 4D,F). Pulmonary thrombosis did not alter tumor apoptosis in WT or conditional KO mice but did approximately double the levels pulmonary tumor proliferation and vascularisation, and these microbead-induced increases were absent in myeloid HIF1α KO mice and reduced in myeloid HIF2α KO mice (Figure 5A–I). In terms of the tumor composition of myeloid cells, the induction of pulmonary thrombosis did not alter neutrophil content or macrophage content in the WT or conditional KO mice (data not shown). These data together suggest that pulmonary thrombosis increases lung tumorigenesis via the pro-angiogenic activity of HIF1 and HIF2 in myeloid cells.

### 3.4. Indirect Evidence for Paracrine Regulation of Thrombosis-Induced Tumorigenesis by Myeloid HIF-Induced TP

**Figure 6:**
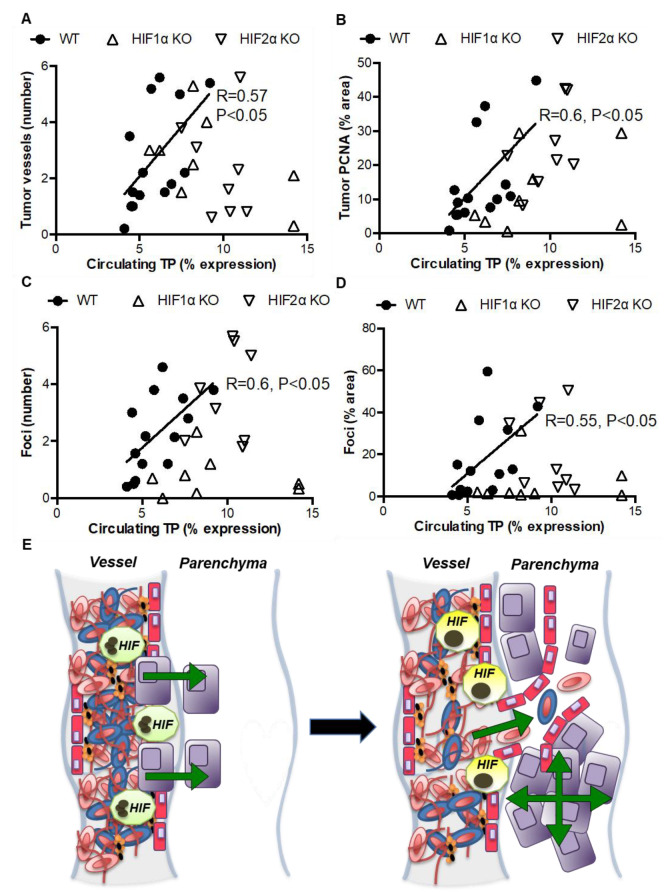
Circulating TP expression is correlated with tumorigenesis in WT but not myeloid HIF1α KO or myeloid HIF2α KO mice. (**A**) Positive correlations between circulating levels of TP and tumor vascularisation, (**B**) proliferation, (**C**) number, and (**D**) area in WT but not myeloid HIFα KO mice at day 14 post-LLC injection. (**E**) Our data suggest that HIF1 and HIF2 signaling is activated in neutrophils (green) and macrophages (yellow) following pulmonary thrombus formation, resulting in enhanced cancer cell (purple) migration and proliferation, and increased lung tumor vascularization and formation.

Given that TP levels were increased in the supernate of neutrophils and macrophages following hypoxia (Figure 1A,B) and increased in the circulation of tumor-free and tumor-bearing mice following microbead administration in our previous study [14], we next studied whether myeloid HIFs could modulate lung tumorigenesis via paracrine expression of TP. In the mice described in the studies above, we observed positive correlations between the circulating levels of TP and tumor vascularisation, proliferation, number, and area in WT mice, but all of these correlations were absent in myeloid HIF1α KO or myeloid HIF2α KO littermates (Figure 6A–D). These data provide indirect evidence that thrombosis- and myeloid HIF-dependent increases in pulmonary tumorigenesis could occur via increases in the paracrine expression of TP.

## 4. Discussion

Thrombosis can promote tumor progression by providing a scaffold for cancer cell attachment and trans-endothelial migration, a binding site for pro-tumorigenic factors, and protection of tumor cells from natural killer cell-mediated death [6,32,33]. Here, we show that hypoxia enhances the levels of multiple tumorigenic factors in myeloid cell supernates, and that these supernates increase tumor cell migration and proliferation in a myeloid HIF-dependent manner. We also show that myeloid HIF1α or HIF2α deletion abolishes the thrombosis-induced increases in pulmonary tumor formation and that myeloid HIF-dependent increases in tumor burden following pulmonary occlusion are associated with increases in tumor proliferation and vascularisation. Intriguingly, we also found that hypoxia increases the expression of TP in supernates of neutrophils and macrophages, while in mice, circulating levels of this factor are positively correlated with tumor propagation in WT but not myeloid HIF1α KO or myeloid HIF2α KO mice. We therefore speculate that pulmonary thrombosis enhances tumorigenesis via myeloid HIF-dependent release of tumorigenic factors such as TP, which promote cancer cell migration and proliferation (Figure 6E). This possibility is supported by previous demonstrations that: (i) murine venous thrombosis increases the circulating levels of tumorigenic factors; (ii) HIF1α is induced in neutrophils contained within occlusive murine venous thrombus, while HIF2α is induced in macrophages contained within propagating murine venous thrombus; (iii) macrophages and neutrophils express multiple tumorigenic factors under hypoxia (including TP); and (iv) pharmacological upregulation of HIF1α in thrombosed mice increases the local expression of multiple tumorigenic HIF targets, while pharmacological inhibition of HIF1α accumulation supresses this local tumorigenic drive [9,15,18,24,25].

Endothelial deletion of HIF1α reduced pulmonary tumor formation, while deletion of endothelial HIF2α had the opposite effect in a previous study [23]. The opposing effects of endothelial HIF1α versus endothelial HIF2α deletion shown previously are comparable to the opposing effects of myeloid HIF1α versus myeloid HIF2α deletion on tumorigenesis in this study, which warrants further investigation. These comparable effects could, for example, be regulated via a common nitric oxide-mediated pathway [23,34]. In other words, HIFs could promote or inhibit tumor progression in an isoform-dependent manner, which may be dependent on level of endothelial nitric oxide production [23]. It remains to be seen whether other distinct or common gene targets of myeloid HIF1 versus myeloid HIF2 are responsible for the increase in tumorigenesis seen following pulmonary thrombus formation and myeloid HIF stabilization. In a murine intestinal tumor model, myeloid HIF1α deletion has been shown to reduce tumor formation, likely in a non-canonical and hypoxia-indpendent manner [19]. HIF1α regulates macrophage function while HIF1α in tumor-associated macrophages support tumor growth [19]. Investigations of canonical and non-canonical mechanisms by which pulmonary thrombosis leads to increases in myeloid HIF signaling and downstream increases in tumorigenesis could be important in the development of novel treatments for patients with thrombosis-associated cancer.

Tumor-associated neutrophils and macrophages express many pro-tumorigenic factors, including TP, which is a hypoxia-responsive factor that stimulates tumorigenesis through increases in angiogenesis and tumor cell proliferation [28,35,36,37,38]. Plasma TP levels are increased in patients with liver carcinoma compared with normal individuals, and in those with advanced versus early stage disease [39]. There are also associations between levels of both major HIF isoforms and TP expression in cancer patients, which vary according to the tumor type studied [30,40,41,42,43]. Furthermore, macrophage-specific expression of TP is positively correlated with tumor vascularity and size, and negatively correlated with survival in invasive human breast tumors [28]. Also, in humans, TP expression is positively correlated with vascular density and proliferation in solid cervical tumors, and negatively correlated with patient prognosis in cervical, colon, and oesophageal tumors [36,44]. Given these clinical findings, along with experimental studies [44,45], specific inhibitors of TP are being developed for use in cancer patients, and have entered clinical trials [38,46,47]. Our results provide indirect support for the pro-tumorigenic function of TP, and for the potential development of novel therapies that target TP.

Current and previous data support the possibility that pulmonary thrombosis leads to the stabilisation of myeloid cell HIFs, which in turn increase the circulating expression of multiple tumorigenic factors. Additional studies of different cancer types could help further elucidate the role(s) of cell-specific HIFs in thrombosis-induced tumor progression. In summary, we have demonstrated that pulmonary occlusion-induced thrombosis enhances lung tumorigenesis in a myeloid HIF1α- and myeloid HIF2α-dependent manner. Studies of the mechanisms that regulate thrombosis-induced cancer progression could lead to the development of novel treatments for cancer patients with thromboembolic disease.

## Data Availability

The data presented in this study are available on request from the corresponding author.
